# Brain state before a memory probe and associative retrieval in older adults

**DOI:** 10.1016/j.neurobiolaging.2018.04.001

**Published:** 2018-08

**Authors:** Jiangyi Xia, Giulia Galli, Leun J. Otten

**Affiliations:** Institute of Cognitive Neuroscience, University College London (UCL), London, UK

**Keywords:** Source memory, Retrieval, EEG, Brain states, Anticipation, Individual differences

## Abstract

Older individuals' difficulty in remembering events from a particular time and place may be explained by changes in retrieval-related control processes. We investigated how aging affects neural activity leading up to a retrieval probe and how such activity relates to later performance. Electrical brain activity was recorded while healthy younger and older humans memorized visual word pairs consisting of an object word (e.g., doll) preceded by a location word (e.g., garden). Only object words were presented during the memory test, the task being to decide whether an object had been presented earlier and, if so, what location had been paired with it. A warning signal before each test probe alerted to an upcoming object. A sustained negative-going event-related potential deflection preceded objects whose associated location could be remembered, especially in older individuals. The poorer an older individual's associative memory, the bigger was this deflection. Aging thus seems accompanied by changes in anticipatory brain states that relate to recollection. Such states may serve to mobilize control processes that aid the recovery of contextual details.

## 1 Introduction

Healthy aging is generally associated with a decline in memory abilities, predominantly in the intentional recollection of specific events (Light, 1991). The most robust age-related memory impairment is in remembering contextual details associated with an event, that is, episodic memory. Successful retrieval of specific contextual details often benefits from cognitive control that modulates retrieval processes in a goal-directed manner (Rugg and Wilding, 2000). In aging, cognitive impairments are often linked to a decline in executive control functions (West, 1996), which suggests that episodic memory deficits in aging may be influenced by changes in cognitive control at retrieval.

An important form of cognitive control is the ability to engage anticipatory mechanisms to optimize performance on the basis of predictions formed from the environment. In memory encoding, it has been shown that older adults are impaired in the ability to use predictive cues to benefit encoding of an item (Bollinger et al., 2011), which may reflect a general expectation deficit in aging (Zanto et al., 2011). In memory retrieval, parallel evidence of an effect of aging on anticipation is lacking. Existing evidence shows that in young adults, cueing subjects to retrieve different kinds of information from memory leads to alterations in anticipatory brain activity before the presentation of a retrieval probe (Johnson and Rugg, 2006). Such preprobe brain activity may reflect an initial task set configuration (Herron and Wilding, 2006) or a preactivation of memory representations of the targeted information (Polyn et al., 2005). Crucially, it has been demonstrated that preprobe anticipatory activity can relate to subsequent retrieval success (Addante et al., 2011).

The idea that preprobe brain activity may reflect an important aspect of retrieval has mostly been neglected in aging studies. Only one study has to our knowledge compared preprobe activity in older and younger adults (Dew et al., 2012). Age differences were seen in brain activity related to general preparation for contextual retrieval, but such activity was not found to be relevant for subsequent retrieval success. Although the study relied on a hemodynamic neuroimaging technique and therefore had reduced ability to pinpoint brain activity in time, the findings are suggestive of an effect of aging on mechanisms that precede a retrieval probe. Further evidence for this idea comes from research on retrieval orientation, that is, the adoption of goal-directed retrieval sets. For old/new recognition, retrieval orientation has been found to be sensitive to aging (Duverne et al., 2008, Morcom and Rugg, 2004). No age-related differences have been observed for contextual retrieval (Duverne et al., 2008), but retrieval orientation was in those studies measured indirectly with postprobe, rather than directly via preprobe, brain activity.

The aim of the present study was to assess the role of aging in brain activity that precedes the retrieval of contextual details. The question was 2-fold: (1) whether the ability to retrieve contextual details relates to brain activity that precedes a retrieval probe and (2) how any such activity is affected by aging. Brain activity was measured via electroencephalography (EEG) to take advantage of its high temporal resolution to isolate retrieval effects across age before a retrieval probe. Older and younger participants saw a series of object words (e.g., doll), each preceded by a location word (e.g., garden). The location served as the to-be-retrieved context, with each object word paired with unique associative information. Participants were asked to judge whether it was easy or difficult to imagine the object in that particular location. During retrieval, participants were shown a mixture of studied and unstudied objects and judged whether each object was old or new. If the object was old, they were asked whether they could remember information associated with the object and report that information verbally. Crucially, each object in the retrieval task was preceded by a warning signal, which alerted participants to the imminent presentation of the memory probe. The key question was whether brain activity in response to the warning signal differed between younger and older adults depending on whether associative information could later be recollected.

## 2 Materials and methods

### 2.1 Participants

Twenty-six younger volunteers were recruited from the University College London student community and 28 older volunteers from the local community and University of the Third Age. Volunteers were remunerated at £7.5/h for participation in the experiment. Two younger and 4 older volunteers were excluded from the analyses because of inadequate memory performance or poor EEG quality leading to insufficient trial numbers (see Section 2.5, for exclusion criteria). The final groups comprised 24 older adults (mean age 67.3 years, range 60–79 years, 12 men) and 24 younger adults (mean age 22.7 years, range 19–29 years, 10 men). All participants were screened via email or telephone to ensure that they were native English speaking, right-handed, had normal or corrected-to-normal vision, did not have a history of neurological or psychiatric conditions, and were not taking psychotropic medication. The experimental procedures were approved by the University College London research ethics committee. All participants provided written informed consent before participating.

### 2.2 Tasks

The experiment used an associative memory paradigm in which participants initially memorized pairs of words. On each trial, an object word (e.g., doll) was preceded by a location word (e.g., garden) that served as the to-be-retrieved associative information in a later memory test. Participants were instructed to create an association between the 2 words by imagining the object in the location and press 1 of 2 buttons depending on whether the association was easy or difficult to make. Participants were told that the decision was subjective and that the task was designed to help them remember word pair associations. This task was followed by a memory test in which all object words were presented again, intermixed with new object words. For each object, participants had to decide whether the object had been presented earlier and, if so, what location had been paired with it. One of 4 buttons had to be pressed depending on whether the location associated with the object was recollected, information associated with the object other than the location was recollected, no associative information was recollected, or the object was not recognized as having occurred earlier. Participants verbally reported any associative information they recalled.

Crucially, a neutral warning signal was presented before each test probe to signal the imminent arrival of an object about which a decision had to be made. The primary interest was whether the neural activity elicited by the warning signal differed depending on whether the location-object association could be recollected and whether this was the case for older and younger adults.

### 2.3 Stimulus material

The tasks were constructed from a pool of 430 words that were between 3 and 8 letters in length and had a written frequency between 1 and 350 per million (Wilson, 1988). Of these words, 172 depicted a physical location (e.g., “garden” or “school”) and 258 an object, half of which were living (e.g., “pigeon”) and half nonliving (e.g., “hammer”). Object words were randomly allocated to 3 sets of 78 each with the constraint that equal numbers of living and nonliving objects were present in each set. Two of the sets were used to create a study list of 156 object words, each randomly paired with a location word. All 3 sets were combined to create a test list of 234 object words, consisting of the 156 object words that had occurred in the study list and 78 new object words. The sets were rotated across participants such that each object occurred as an old or new item, and new random sequences were generated for each participant. The remaining object and location words were used to create practice lists.

### 2.4 Procedure

Participants were tested individually inside a quiet chamber. An experimental session started with the application of the EEG cap. The participant then read and verbally described the task instructions before performing short practice sessions until they fully understood and felt comfortable with the tasks. Data were acquired during 6 study-test blocks. Pilot work indicated that this would allow sufficient numbers of location-object pairs to be remembered and forgotten in both age groups to compute event-related potentials (ERPs). Each study phase contained 26 location-object word pairs, followed by a test phase containing the 26 studied and 13 unstudied object words. The test phase started without delay once the participant was ready to proceed.

All stimuli were presented visually in white uppercase Helvetica letters (font size 30) against a gray background on a computer monitor. Participants were instructed to focus their eye gaze on the center of the screen, blink normally, and avoid muscle tension while performing the tasks to minimize artifacts in the EEG. Each study trial began with the presentation of a warning signal (a red exclamation mark) for 1.9 seconds, followed by a blank screen for 100 ms, a location word for 1.5 seconds, a fixation cross for 500 ms, and then an object word for 1.5 seconds. The next trial followed after a variable interval between 3 and 4.5 seconds. Participants indicated whether the association between the location and object was easy or difficult to make by pressing 1 of 2 buttons with the left or right middle finger as quickly yet accurately as possible. Responding hand was counterbalanced across participants.

In the test phase, each trial again began with the presentation of a warning signal (a red exclamation mark) for 1.9 seconds, followed by a blank screen for 100 ms. An object word was then presented for 1.5 seconds, which required participants to decide whether the object was old or new and whether they could remember the source location. One of 4 buttons, representing the “old/location”, “old/other information”, “old/no information,” and “new” categories, was pressed with the index and middle fingers of both hands. The “old/other information” option was included to ensure that the comparison between association-remembered trials and association-forgotten trials would not be contaminated by the recollection of associative information other than the location (Galli and Otten, 2011).

There was no time limit for the button presses, although both accuracy and speed were stressed. This self-paced design was adopted to accommodate the variability in response time common in older adults and to maximize the accuracy of their retrieval. When a “new” or “old/no information” response was made, a fixation cross was presented for 2 seconds before the onset of the next trial. When an “old/location” or “old/other information” response was made, the fixation cross was presented for a further 4 seconds while participants verbalized the location or the other information they recalled. Response assignments for the keys associated with study and test decisions were counterbalanced across participants. Stimulus presentation and behavioral response recording were controlled using MATLAB software on Windows PCs.

After all 6 study-test blocks had been completed, participants were asked about the way they had completed the tasks. The EEG cap was then removed, and participants relaxed while having their hair washed. The EEG session, including application of the electrode cap and running of the task (excluding the neuropsychological testing), lasted approximately 2.5 hours for younger participants and 3 hours for older participants, as it took longer for the older participants to learn and complete the tasks.

At the end of the experimental session, participants were administered a battery of 11 neuropsychological tests to assess a range of cognitive functions that may decline with age. The Mini–Mental State Examination was used to screen against dementia (Folstein et al., 1975) with a cutoff score of 26/30. Long-term memory was assessed with the Verbal Paired Associates and the Word List (Immediate and Delayed) from the Wechsler Memory Scale-III. Short-term memory was assessed with the forward and backward Digit Span test. General cognitive functions such as processing speed and executive function were assessed using the Trail Making tests A and B and the verbal fluency tests (letter and category), respectively. The second edition of the National Adult Reading Test was used to obtain an estimation of full-scale IQ. The Geriatric Depression Scale was also administered to both age groups.

### 2.5 EEG acquisition and preprocessing

**Fig. 1:**
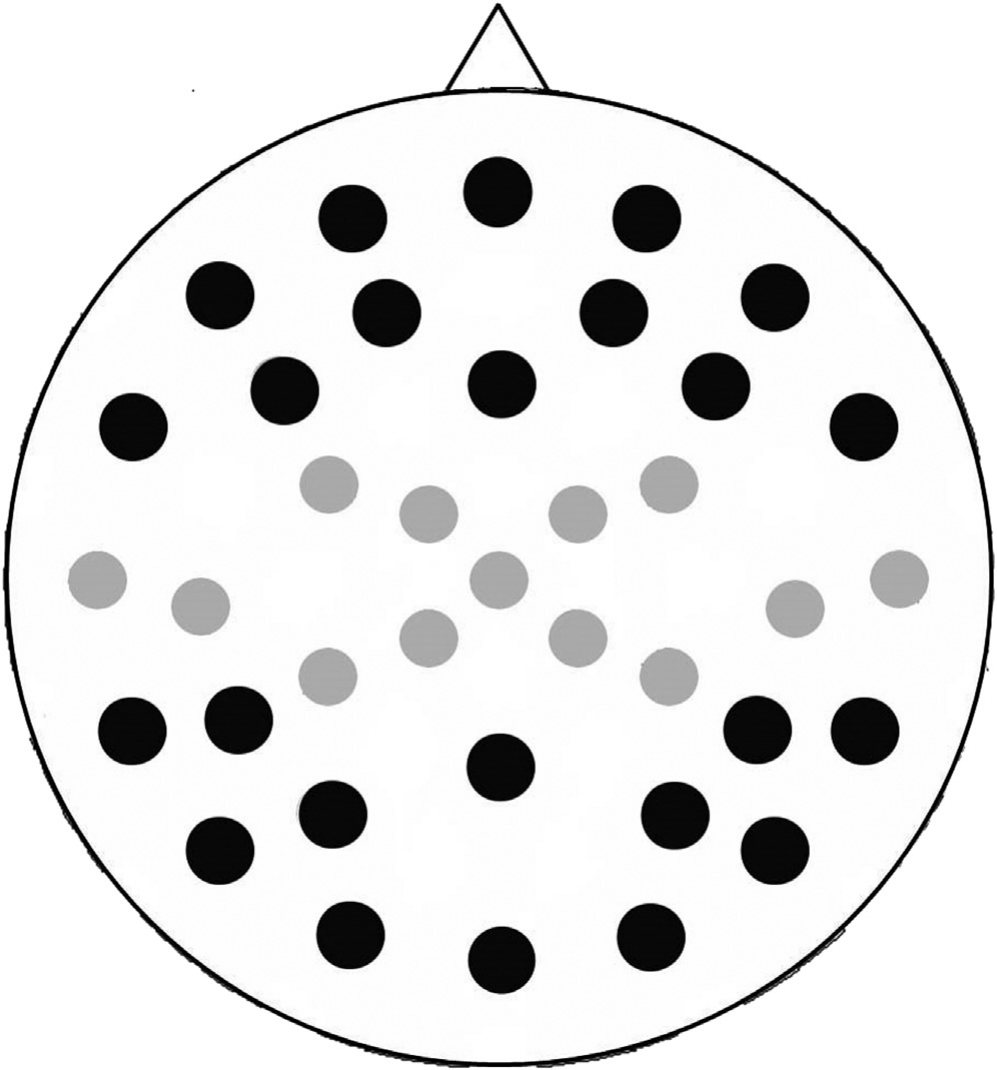
Schematic illustration of all 37 recording scalp sites. The 12 anterior and 12 posterior sites included in the statistical analyses are shown in black.

EEG was recorded with sintered silver/silver-chloride electrodes from 37 scalp sites using Easycap montage 10 (www.easycap.de/e/electrodes/13_M10.htm; Fig. 1), referenced online to a midfrontal site corresponding to Fz in the 10/20 system. Vertical and horizontal eye movements were recorded bipolarly from electrodes above and below the right eye and at the outer canthus of each eye, which were used to correct trials contaminated by eye-movement artifacts. Impedance was tested and reduced to below 5kΩ before recording. On-line, signals were amplified, bandpass-filtered between 0.01 and 35 Hz (3 dB roll-off), and digitized at 500 Hz with 12-bit resolution. Off-line, the data were digitally filtered between 0.05 and 20 Hz (96 dB roll-off, zero phase shift filter).

Neural activity elicited by the warning signals during retrieval, that is, preprobe anticipatory brain activity, was extracted and segmented into 2560 ms epochs, starting 100 ms before the onset of the warning signals. The data were downsampled to 100 Hz to accommodate a limitation in the analysis software that each epoch can only contain 256 sample points. ERPs were computed from these epochs for each participant at each electrode site for stimuli later given different responses. ERPs were baseline corrected to the 100 ms before the onset of the warning signal. Trials with nonblink eye-movement artifacts, muscle tension, analog-to-digital saturation, and drifts exceeding ±50 μV were excluded from the averaging process. Blink artifacts were minimized via estimating and correcting their effects using a regression method (Rugg et al., 1997). The data were then re-referenced to an average mastoid reference (re-instating the online reference Fz as a site of interest).

Grand-average ERPs were derived for each electrode site by averaging across participants according to response categories. To ensure a reasonable signal-to-noise ratio, a minimum of 16 artifact-free trials per condition was set as the criterion for a subject to be included. For the critical contrasts between associative hits and misses, 1 older adult was excluded due to insufficient (<16) artifact-free trials. EEG data were thus taken from 23 older and 24 younger participants. For these participants, the average numbers and ranges (in parentheses) of artifact-free trials for associative hits and associative misses were 43 (18–111) and 57 (23–107) for the older group and 81 (44–122) and 39 (16–70) for the younger group, respectively.

### 2.6 Experimental design and statistical analysis

#### 2.6.1 ERP analyses

The experiment was designed to contrast neural activity before a test probe, in the interval between the warning signal and probe, depending on whether the probe gave rise to successful recollection of the location-object pairing. The crucial experimental contrast in this respect was within participants, between associative hits and associative misses in the memory task. Associative hits refer to trials on which old objects were recognized as being old and for which the location associated with the object during the study phase could be recollected (“old/location” responses with correct location verbally reported). Associative misses refer to trials on which old objects were recognized as being old but for which the associated location was forgotten (“old/no information” response). Retrieval-related anticipatory activity was quantified on these trials by computing mean amplitude values in the ERP waveforms in 2 equal latency intervals: 200–1100 and 1100–2000 ms after onset of the warning signal. These intervals are in line with previous work on anticipatory processes in younger adults (Galli et al., 2011, Gruber and Otten, 2010, Johnson and Rugg, 2006). The paucity of research on aging and anticipatory activity prevented an adjustment of these intervals for older individuals, but because age was expected to primarily affect ERP amplitude, the chosen latency intervals should capture effects in both age groups.

The mean amplitude values were submitted to a mixed-model analysis of variance (ANOVA) incorporating the between-participants factor of age (older, younger) and within-participants factors of memory (associative hit, associative miss), interval (200–1100, 1100–2000 ms), and scalp location (anterior, posterior). Statistical analyses were conducted on 24 electrode sites (Fig. 1) to characterize scalp distribution according to anteriority (anterior/posterior) given the expectation that preprobe effects would be largest over frontal scalp sites (Herron and Wilding, 2006, Johnson and Rugg, 2006). The analyses contrasted activity on the 12 most anterior scalp sites with that on the 12 most posterior ones; differences within the 12 anterior and 12 posterior sites were not of experimental interest and therefore not considered. If retrieval-related activity differed across latency intervals, subsidiary ANOVAs were conducted for each interval separately. The Greenhouse-Geisser correction for nonsphericity was used when appropriate and degrees of freedom were adjusted accordingly.

Possible differences in the way younger and older adults generally prepared for a retrieval attempt, irrespective of later retrieval success and old/new status of the test probe, were evaluated by collapsing ERP waveforms across all artifact-free trials (cf. Dew et al., 2012). For these analyses, the data were collapsed across the 200–2000 ms interval after the warning signal and activity was considered across all electrode sites because no pre-experimental predictions existed as to the nature of any effects that might be observed. The mixed-model ANOVA accordingly incorporated the between-participants factor of age (older, younger) and within-participants factor of scalp location (all 37 sites). Any general preparatory effect was planned to be compared with retrieval-related anticipatory activity to assess the difference or similarity between the two.

Analyses of scalp distribution were performed where appropriate to determine whether ANOVA interactions involving scalp site were due to overall amplitude differences between groups/conditions or a true reflection of differences in topography (McCarthy and Wood, 1985). ERP data were rescaled using the max/min method. For effects involving memory, rescaling was applied to difference scores (associative hit–associative miss) for each age group including all 37 electrodes. Although rescaling is not uniformly considered to be necessary (e.g., Keil et al., 2014), this approach provides a conservative estimate of possible scalp distribution differences across effects of interest.

#### 2.6.2 Behavioral analyses

In addition to neural activity, several aspects of participants' behavior during study and test were of interest. Performance on the study task was analyzed to examine whether the 2 age groups differed in their subjective judgments about how difficult it was to associate the object and location words, and the amount of time they took processing easy and difficult pairs. Mixed-model ANOVAs were performed on the proportions of easy and difficult responses and response times, incorporating the between-participants factor of age (older, younger) and within-participants factor of difficulty (easy, difficult).

The analyses of the data from the memory test focused on participants' ability to recollect associative information. To that end, the proportion of associative hit trials relative to all trials on which an old object was correctly recognized was computed. These values were entered into an independent samples *t* test, comparing the 2 age groups. The response times associated with associative hits and associative misses were also considered. These were contrasted in a mixed-model ANOVA with a between-participants factor of age (older, younger) and a within-participants factor of memory (associative hit, associative miss).

The ability to discriminate between old and new objects, irrespective of the retrieval of associative information, was also assessed for the behavioral data. Measures of discrimination accuracy (Pr = H − FA) and response bias [Br = FA/(1 − Pr)] were computed, where “H” refers to the proportion of hits (collapsed across “old/location”, “old/other information”, and “old/no information” responses) and “FA” to the proportion of false alarms (Snodgrass and Corwin, 1988). These measures were contrasted in a mixed-model ANOVA with the between-participants factor of age (older, younger). Item recognition could not be analyzed for the neural data as pilot work was done purposely to foster a high level of recognition for the retrieval probes. This ensured that sufficient numbers of trials were generated for both associative hits and associative misses, but at the expense of trial numbers for old items misclassified as new.

Finally, between-participants *t* tests were performed on years of education and neuropsychological test scores to examine possible ability differences between age groups. Type I errors may affect the outcome of these analyses given the number of tests that were performed (12). The Bonferroni approach was therefore adopted to correct for the multiple comparison problem, lowering the required significance level for each comparison to 0.004 (0.05 divided by 12).

#### 2.6.3 Participant number

The number of participants included in the experiment was based on previous work on anticipatory influences on memory retrieval, primarily in younger adults. Because virtually no work exists on age-related differences, it was not possible to compute a meaningful statistical power analysis based on an expected effect size. The number of participants of 24 in each group exceeded the numbers used in previous studies (Addante et al., 2011, Dew et al., 2012, Morcom and Rugg, 2004) and was therefore considered acceptable to establish age-related differences in anticipatory activity.

## 3 Results

### 3.1 Neuropsychological test scores

Compared to younger adults, older adults showed a typical pattern of age-related impairments and preservations (Table 1). This included lower scores on tests for long-term memory, processing speed, and executive functions, higher scores on tests for crystallized intelligence, and similar scores on tests for short-term memory. All participants scored 27 or above on the Mini–Mental State Examination.

**Table 1:** Participant characteristics and neuropsychological test scores for the 2 age groups

Participant characteristic and neuropsychological test	Older group		Younger group		*p*
	Mean (SD)	Range	Mean (SD)	Range	
Years of education	15.1 (3.1)	9–20	16.7 (2.4)	13–22	0.044
MMSE	29.0 (0.9)	27–30	29.5 (0.9)	27–30	0.059
Verbal Paired Associates	22.0 (7.9)	6–32	28.9 (2.8)	20–32	<0.001^a^
Word List (Immediate)	33.8 (7.1)	22–45	41.4 (4.3)	33–48	<0.001^a^
Word List (Delayed)	8.5 (2.9)	3–12	10.3 (1.7)	5–12	0.011
Digit Span (Backward and Forward)	20.5 (4.2)	14–30	22.3 (4.0)	16–28	0.148
Verbal Fluency–Letter	43.5 (12.6)	23–89	46.6 (9.4)	30–67	0.340
Verbal Fluency–Category	28.5 (6.4)	22–48	37.5 (5.0)	29–47	<0.001^a^
Trail Making Part A (s)	36.2 (9.2)	21–54	22.6 (6.7)	11–38	<0.001^a^
Trail Making Part B (s)	89.2 (34.7)	34–199	56.2 (21.3)	23–114	<0.001^a^
NART (FSIQ estimate)	119.0 (6.8)	105–131	110.8 (6.5)	95–122	<0.001^a^
GDS (short form)	3.0 (3.8)	0–13	2.7 (4.0)	0–15	0.770

Key: FSIQ, full-scale IQ; GDS, Geriatric Depression Scale; MMSE, Mini-Mental State Examination; NART, National Adult Reading Test; SD, standard deviation.

a Remains significant after Bonferroni correction.

### 3.2 Task performance

In the study task, the 2 age groups had similar opinions on the ease with which objects could be imagined in the paired locations (Table 2). The 2 (age: older, younger) by 2 (difficulty: easy, difficult) mixed-model ANOVA on trial proportions showed a significant effect of difficulty [*F* (1, 46) = 8.79, *p* = 0.005, *η*_*p*_^*2*^ = 0.160], with more pairs judged as difficult to link, but no age by difficulty interaction. For the same ANOVA on response times, there was a significant main effect of age [*F* (1, 46) = 13.65, *p* = 0.001, *η*_*p*_^*2*^ = 0.229], but no age by difficulty interaction or main effect of difficulty. Thus, while older adults were on the whole slower in responding, response times within each group did not differ between object-location pairs that were easy and difficult to link.

**Table 2:** Proportions of object-location pairs judged easy or difficult to link by the 2 age groups and associated response times

Age group	Proportion (standard deviation)		Response time in ms (standard deviation)	
	Easy	Difficult	Easy	Difficult
Older	0.41 (0.22)	0.57 (0.21)	2586 (986)	2620 (1099)
Younger	0.40 (0.18)	0.59 (0.18)	1736 (522)	1745 (486)

Aging affected the ability to recollect the locations paired with objects (Table 3). An independent samples *t* test on the proportions of recognized old items for which the associated location could be retrieved, relative to all recognized old items, showed a significant difference between age groups [*t* (45) = 6.01, *p* <0.001, *d* = 1.75]. Younger adults were able to recollect the associated location more frequently than older adults (62.7% vs. 39.3%, respectively). The associated response times were contrasted in a mixed-model ANOVA with a between-participants factor of age (older, younger) and a within-participants factor of memory (associative hit, associative miss). This ANOVA showed significant main effects of age [*F* (1, 46) = 10.62, *p* = 0.002, *η*_*p*_^*2*^ = 0.188] and memory [*F* (1, 46) = 24.90, *p* <0.001, *η*_*p*_^*2*^ = 0.351], but the interaction between the two was not significant (*p* = 0.475). As expected, older adults were slower to respond than younger adults irrespective of response category, and both age groups were faster when associative information could be retrieved. The similarity across age groups in the pattern of response times is important for the present investigation because it indicates that any between-group differences in neural correlates of associative retrieval are unlikely to reflect mere differences in response times.

**Table 3:** Associative memory performance in the 2 age groups

Age group	Hit, location recalled	Hit, other information recalled^a^	Hit, no associated information recalled	Miss	Correct rejection
Proportion of responses (standard deviation)					
Older	0.35 (0.16)	0.03 (0.04)	0.46 (0.15)	0.12 (0.07)	0.89 (0.10)
Younger	0.60 (0.11)	0.04 (0.05)	0.29 (0.12)	0.05 (0.03)	0.93 (0.08)
Response time in ms (standard deviation)					
Older	3177 (1246)	5674 (3718)	4010 (2051)	2505 (880)	1731 (519)
Younger	1749 (498)	3750 (2532)	2863 (1818)	2111 (988)	1263 (435)

Hits with incorrect recalled locations are excluded from the table.

a Seven older and 5 younger participants did not have any trials in this category and were therefore excluded from the computation of response times in this category.

Aging also affected the ability to discriminate between old and new objects irrespective of whether the paired location could be retrieved. Discrimination accuracy (Pr) was 0.77 (standard deviation [SD] = 0.13) for older adults and 0.89 (SD = 0.08) for younger adults. A between-subjects *t* test indicated that this difference was statistically significant [*t* (46) = 3.81, *p* <0.001, *d* = 1.10]. The 2 age groups did not differ in response bias (Br), which was 0.41 (SD = 0.23) in younger adults and 0.41 (SD = 0.33) in older adults (*p* = 0.943).

### 3.3 ERPs

**Fig. 2:**
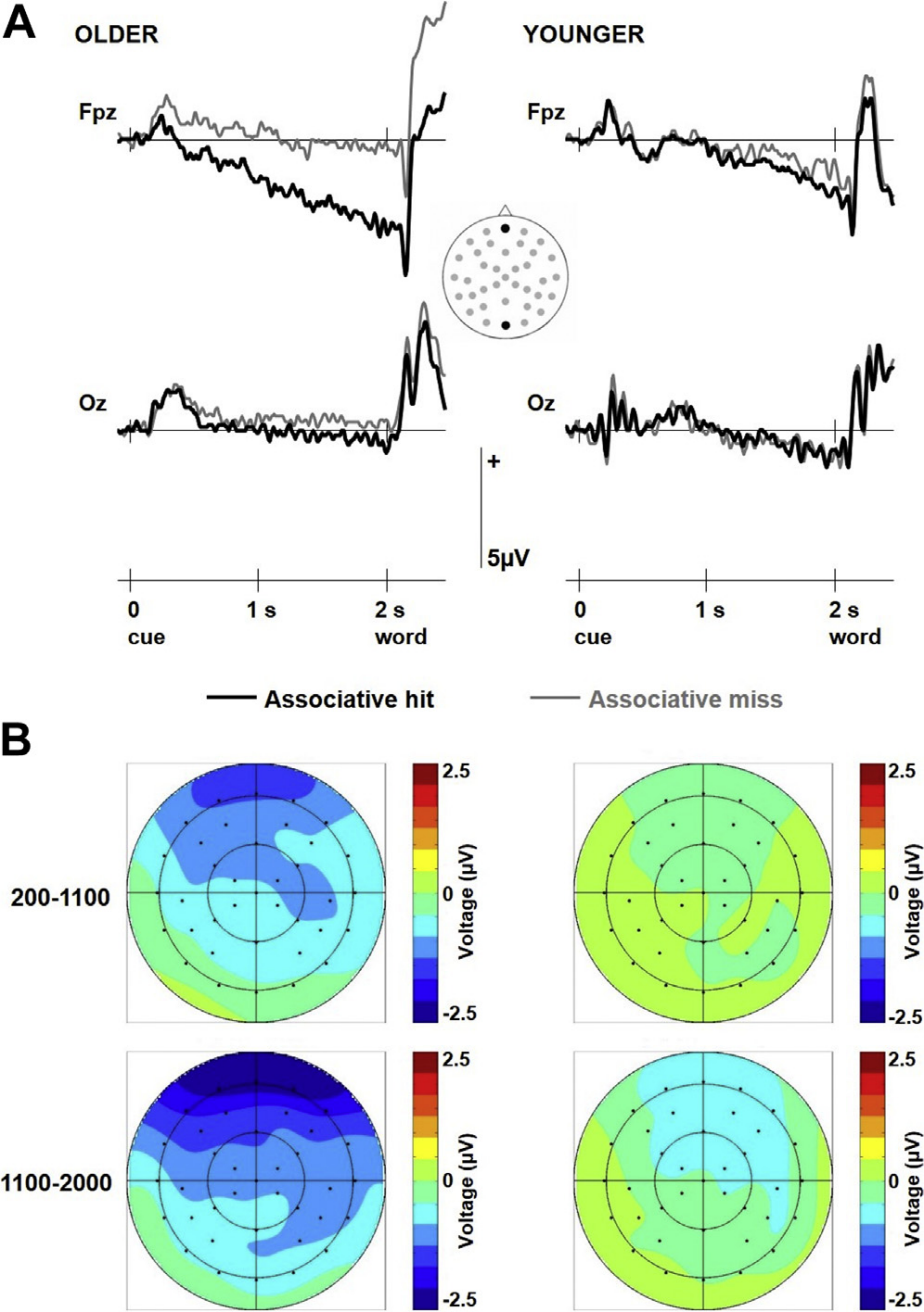
Preprobe brain activity related to associative retrieval. (A) Grand-average ERPs for the 2 age groups elicited by warning signals that indicated the imminent presentation of a retrieval probe. ERPs are shown separately for trials on which the probe did versus did not elicit the retrieval of the location associated with the recognized old object. The insert indicates the locations of the 2 electrodes that are shown (site 35 and 43 from Montage 10; www.easycap.de/e/electrodes/13_M10.htm; equivalent to site Fpz and Oz of the 10-20 system, respectively). (B) Voltage spline maps for the older and younger groups showing the distribution of the difference between brain activity preceding associative retrieval and no associative retrieval in the 200–1100 ms and 1100–2000 ms intervals after the warning signal. The maps are symmetrically scaled to the same range across intervals and groups. Abbreviation: ERPs, event-related potentials.

During the memory test, ERP waveforms for older adults diverged soon after the onset of the warning signal depending on whether associative information was later recollected (Fig. 2). The effect was negative-going and built up gradually during the interval between the warning signal and memory probe, reaching its maximum just before probe onset. The effect showed a widespread distribution with a maximum at anterior scalp sites. In younger adults, a similar effect was seen but it started later, at around 1100 ms after the warning signal, and was much attenuated in size.

The mixed-model ANOVAs on the mean amplitude values in the 200–1100 and 1100–2000 ms intervals after the warning signal revealed a main effect of memory [*F* (1, 45) = 8.08, *p* = 0.007, *η*_*p*_^*2*^ = 0.152], modulated by interactions with group [*F* (1, 45) = 4.44, *p* = 0.041, *η*_*p*_^*2*^ = 0.090], location [*F* (1, 45) = 6.78, *p* = 0.012, *η*_*p*_^*2*^ = 0.131], and, importantly, interval [*F* (1, 45) = 5.41, *p* = 0.025, *η*_*p*_^*2*^ = 0.107]. This suggests that retrieval-related activity differed across latency intervals, inviting separate subsidiary ANOVAs for each interval. These revealed that between 200 and 1100 ms after warning signal onset, there was a significant main effect of memory [*F* (1, 45) = 4.40, *p* = 0.042, *η*_*p*_^*2*^ = 0.089] and a memory by group interaction [*F* (1, 45) = 4.38, *p* = 0.042, *η*_*p*_^*2*^ = 0.089]. The interaction corroborated the observation that the preprobe effect was present in older adults [−0.83 μV averaged across scalp sites and time windows: *F* (1, 22) = 6.33, *p* = 0.020, *η*_*p*_^*2*^ = 0.223] but not in younger ones (0 μV: *F* <1). In the 1100–2000 ms interval, a main effect of memory again emerged [*F* (1, 45) = 9.37, *p* = 0.004, *η*_*p*_^*2*^ = 0.172], modulated by an interaction between memory and scalp location [*F* (1, 45) = 7.84, *p* = 0.008, *η*_*p*_^*2*^ = 0.148]. The interaction reflected that the retrieval-related effect was larger over anterior [−1.21 μV: *F* (1, 45) = 11.03, *p* = 0.002, *η*_*p*_^*2*^ = 0.197] than posterior [−0.48 μV: *F* (1, 45) = 4.27, *p* = 0.045, *η*_*p*_^*2*^ = 0.087] scalp locations. No age differences emerged in the later interval, although the memory by group interaction approached significance [*F* (1, 45) = 3.55, *p* = 0.066, *η*_*p*_^*2*^ = 0.073].

**Fig. 3:**
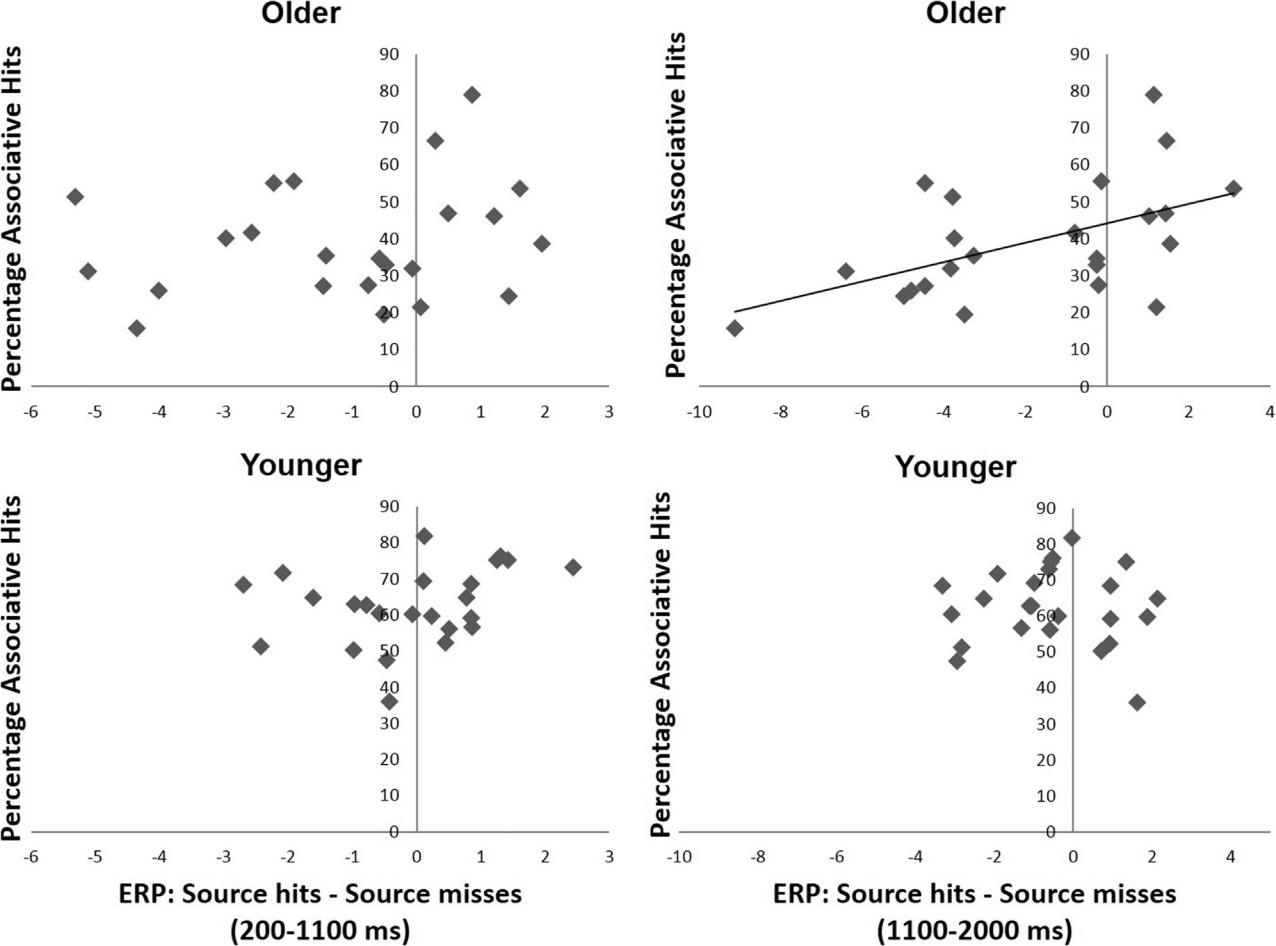
Scatterplots showing the between-subject relationship between the size of an individual's preprobe ERP effect (averaged across the 12 anterior scalp sites) and the individual's overall associative memory performance (the percentage of associative hits relative to all correctly recognized old items). The plots are shown for the older group (top, *N* = 23) and younger group (bottom, *N* = 24) in the early (left, 200–1100 ms) and later (right, 1100–2000 ms) ERP intervals. A significant correlation between ERPs and behavior was found for older but not younger adults in the interval just before probe onset (see Section 3.3). Abbreviation: ERPs, event-related potentials.

Taken together, the data show evidence of a preprobe associative retrieval effect, which started early for older adults and was then found across age groups just before probe onset especially over anterior scalp sites. To assess the functional significance of this effect, across-subject correlations were computed between the size of the preprobe effect and an individual's overall ability to retrieve associative information. Older adults showed more individual variation in ERPs and memory performance than younger adults (Fig. 3), raising the interesting possibility of a relationship between the brain and behavior. Such a correlation would strengthen the conclusion that preprobe activity is important for later memory performance and as a consequence may help understand the functional role of the activity in memory.

For each individual, a measure of the preprobe associative retrieval effect was obtained by taking the difference in ERP amplitudes between associative hits and associative misses in the 200–1100 and 1100–2000 ms intervals, collapsed across the 12 anterior scalp sites. This measure was contrasted with the individual's percentage of associative hits (Fig. 3). For the early ERP interval, no significant Pearson Product Moment correlations were found between measures (older adults: *r* = 0.22, *p* = 0.321; younger adults: *r* = 0.289, *p* = 0.170). For the later interval, however, a significant correlation emerged for older adults (*r* = 0.53, *p* = 0.010), but not for younger adults (*r* = −0.04, *p* = 0.856). A direct comparison across age groups showed that the correlation was indeed only significant for older adults in this interval (Fisher r-to-z transformation, *z =* 2.00, *p* = 0.046).

To address the specificity of this brain-behavior correlation, an additional exploratory analysis was performed in which the preprobe ERP effect was correlated with older individuals' scores on the 2 neuropsychological tests that tapped into aspects of long-term memory (see Section 2.4). These were the Verbal Paired Associates test as a proxy for older individuals' associative memory ability and the Delayed Word Lists test as a proxy for item memory ability. The scores on the former correlated significantly with the ERP preprobe effect (*r* = 0.48, *p* = 0.019, significant after Bonferroni correction), whereas the latter did not (*r* = 0.27, *p* = 0.218).

An important question is whether the preprobe retrieval effect in older individuals may be influenced by speech-related processes on preceding trials. Verbal responses were required when associative information was retrieved, and older adults tended to give later and lengthier answers relative to younger adults. It might be that, proportionally, trials on which the object-location pairing could be retrieved were preceded more often by trials that required verbal responses. To rule out this possibility, ERP waveforms were computed using only trials preceded by nonspeaking trials. The average number of such artifact-free trials in older adults was 34 (range 16–73) for associative hits and 49 (range 18–96) for associative misses. Reassuringly, the resulting pattern of ERP waveforms closely resembled that found including all trials. A within-participants ANOVA incorporating factors of memory and scalp location again revealed main effects of memory [200–1100 ms: *F* (1, 22) = 7.15, *p* = 0.014, *η*_*p*_^2^ = 0.245; 1100–2000 ms: *F* (1, 22) = 7.83, *p* = 0.010, *η*_*p*_^2^ = 0.262] and interactions between memory and location that also reached or were close to significance [200–1100 ms: *F* (1, 22) = 5.19, *p* = 0.033, *η*_*p*_^2^ = 0.191; 1100–2000 ms: *F* (1, 22) = 4.16, *p* = 0.054, *η*_*p*_^2^ = 0.159]. It is thus unlikely that the preprobe effect in older adults can be explained by an influence of speech that carried forward to associative hit trials.

**Fig. 4:**
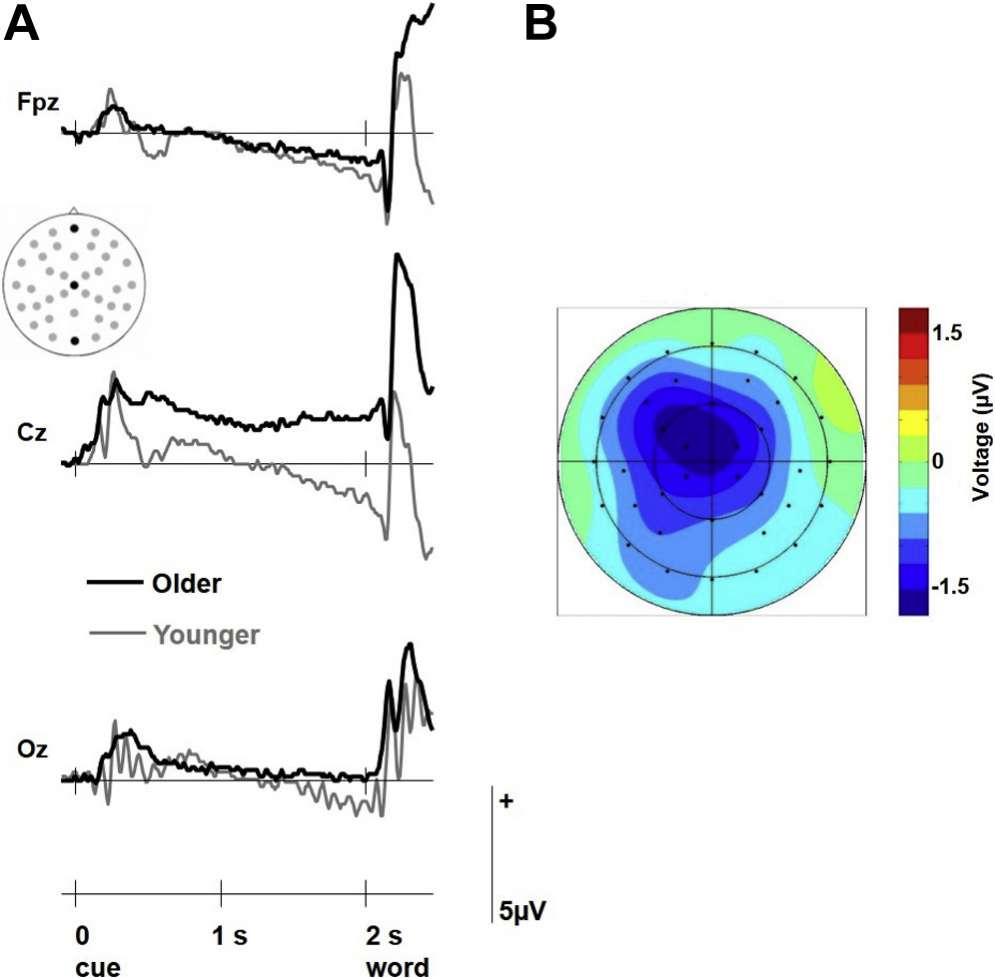
Preprobe brain activity across all trials. (A) Grand-average ERPs for the 2 age groups elicited by the warning signals during retrieval irrespective of the old/new status of the later memory probe or success of the associated memory decision. The insert indicates the locations of the 3 midline electrodes that are shown (equivalent to Fpz, Cz, and Oz of the 10-20 system, respectively). (B) Voltage spline map showing the distribution of the amplitude difference between older and younger individuals in the 200–2000 ms interval after onset of the warning signal. The maps are symmetrically scaled. Abbreviation: ERPs, event-related potentials.

Finally, the analyses assessed possible differences in the way younger and older adults prepared for a retrieval attempt irrespective of retrieval success. Relative to older adults, younger adults showed a markedly more negative-going waveform before probe onset that was widespread across the scalp with a focus over central sites (Fig. 4). This difference resembles the contingent negative variation, a well-known neural signature of perceptual and attentional preparation (Brunia et al., 2012). A mixed-model ANOVA on the data from the 200–2000 ms interval with factors of age (older, younger) and scalp location (all 37 sites) showed significant main effects of age [*F* (1, 45) = 6.12, *p* = 0.017, *η*_*p*_^*2*^ = 0.120] and site [*F* (3.2, 143.8) = 6.50, *p* <0.001, *η*_*p*_^*2*^ = 0.126], and an interaction between the two that just fell short of significance (*p* = 0.059). Thus, although the general preparatory effect was larger in younger individuals, it seemed qualitatively similar across age groups. Crucially, the scalp distribution of this effect differed from that of associative preprobe activity. Whereas the former had a central distribution, the latter was largest over frontal scalp sites. As the preprobe associative memory effect was reliable only in older adults, this observation was verified using a within-participants ANOVA on data from the older group incorporating factors of effect type (general preprobe activity, associative retrieval-related preprobe activity) and scalp site (37 locations). The results showed a main effect of effect type [*F* (1, 45) = 16.44, *p* = 0.001, *η*_*p*_^*2*^ = 0.428], and a significant interaction between effect type and site [*F* (2.9, 62.9) = 3.57, *p* = 0.020, *η*_*p*_^*2*^ = 0.140], which remained significant after scaling [*F* (2.9, 63.7) = 3.20, *p* = 0.031, *η*_*p*_^*2*^ = 0.127]. These results lend support to the idea that retrieval-related preprobe activity differs from general preparatory processes.

## 4 Discussion

The primary interest in this study was whether the brain state just before the onset of a memory probe is relevant for younger and older adults' ability to retrieve associative information from memory. Behavioral analyses showed that, as typically found, younger adults outperformed older adults in the ability to retrieve associative information (Naveh-Benjamin, 2000). These retrieval differences were not accompanied by different patterns of responding during the explicit encoding task. Although older adults were on the whole slower in responding during encoding, they did not differ from younger participants in the relative amounts of time taken to respond to word pairs judged as easy and difficulty. Older and younger participants also did not differ in their subjective judgments about how difficult it was to pair objects with the associated location. During retrieval, the general tendency to respond old or new was equivalent across age groups. The associative memory paradigm used here was thus successful in eliminating the potential confound of response bias in aging research (Huh et al., 2006). With an emphasis on object-location binding, older adults appear to have relied on associative information to guide their responses. The “other associative information” category attracted small but non-zero proportions of responses. This category thus successfully diverted noncritical associative retrieval (the retrieval of associative information other than the paired location) from the associative miss category. Therefore, the contrast between associative hits and associative misses was unlikely to be diluted by the retrieval of different types of associative information. Similarly, differential rates of guessing are unlikely to have contributed to associative hits given that 156 unique object-location pairings were used.

Crucially, the ERP results support the idea that brain activity before a retrieval probe plays a role in the successful retrieval of associative information. The waveforms preceding probes that led to the recovery of associative information were more negative-going than those preceding probes that led to the recognition of an old object but not its associated location. The ERP effect was spatially widespread and largest over frontal scalp sites. It was mostly observed in older adults, particularly in those who performed relatively poorly, and the magnitude of the effect increased with the degree of memory impairment in older participants.

In stark contrast to the present results, a previous study investigating cognitive control during retrieval found that older adults have diminished anticipatory activity relative to younger adults (Dew et al., 2012). Following instructional cues signaling source retrieval, older adults showed reduced activity in the medial temporal lobe coupled with reduced functional connectivity between the left hippocampus and prefrontal cortex. However, the hemodynamic neuroimaging technique only allowed cue-related activity to be measured on trials where the cue was not followed by a memory probe. The relationship between anticipatory activity and retrieval success could thus not be established. In the present study, older and younger adults also differed in overall preparatory activity, but the intracerebral origins of the activity may not match those in the study by Dew et al. (2012). Another possible explanation for the opposite direction of effects is the use of a blocked design in the present study, where the same warning signal was used consistently and participants could maintain a source-related retrieval state across trials. Dew et al. (2012) instead used an intermixed design in which cues that signaled the requirement to retrieve item versus source information varied unpredictably from trial to trial. In that circumstance, preprobe operations have to be reset on a trial-by-trial basis, and older adults may be disadvantaged due to the high switching demands of the task. It may be easier for older adults to engage anticipatory mechanisms in a blocked design, when no additional switch cost is required. Regardless of the precise explanation for the opposite pattern of effects, the current findings are important in that they demonstrate that older adults can use retrieval-related anticipatory processes to a larger extent than younger adults, at least under some circumstances.

What is the functional role of preprobe neural activity in associative retrieval? The gradual increase of the activity in the interval between the warning signal and retrieval probe, with a maximum just before probe onset, suggests that the effect reflects a preparatory process, presumably in service of the upcoming retrieval attempt. The larger activity on source hits may signal a greater degree of preparation on such trials, allowing a more extensive or efficient search of memory that makes successful retrieval of source information more likely. Such a retrieval strategy may especially benefit those individuals whose memory is relatively poor. Clearly, however, the precise role of the preprobe activity cannot be discerned on the basis of the current findings alone. The experiment was designed to be able to investigate associative memory in older and younger individuals, but this came at the expense of not being able to consider item-related or other types of memory. It is thus unknown whether neural activity before probe onset only plays a role in associative memory or may have a more general role, or indeed extend to other cognitive domains. It is suggestive in this respect that the preprobe activity correlated with the neuropsychological test that taps into associative but not item memory, but further work employing a wider range of experimental conditions will be necessary to fully understand the functional role of preprobe neural activity.

None the less, it is important to consider possible functional roles to be able to design future studies and understand the implications of the findings. To do so, extraneous factors that may have affected the results need to be eliminated. One such factor is the differential influence of speech from preceding trials. This is not a likely explanation because the preprobe activity in older adults was still present when the preceding trials did not involve speech. Another possibility is that the activity reflects fluctuations in general alertness. It could be that older adults were unable to maintain an adequate level of attention throughout the test session due to limited resources. This interpretation implies that times of heightened attention preferentially benefitted the retrieval of associative information. However, this idea is difficult to reconcile with the observed dissociation between retrieval-related and general preparatory activity, found irrespective of the old/new status of an upcoming probe. The latter is thought to reflect attentional preparation (Brunia et al., 2012). The fact that a decision and response was required on each trial, expected to require at least a minimum level of alertness, also argues against such an interpretation.

The observed preprobe effect is similar in polarity and scalp distribution to the anticipatory activity seen in encoding paradigms (Otten et al., 2006, Otten et al., 2010). In those studies, frontal negative-going ERP waveforms were predictive of later memory success in tasks requiring semantic processing. The effects were interpreted as goal-directed mobilization of semantic processing resources in anticipation of upcoming semantic decisions. In the present study, the goal was to recall a specific kind of information associated with a retrieval probe, namely a location word. Thus, on the assumption that the preprobe effect seen here during retrieval reflects the same mechanism as during encoding, a plausible account for the preprobe effect is that it reflects the adoption of semantic retrieval strategies that facilitate the recovery of targeted associative information.

Preprobe activity might also modulate retrieval through neural reinstatement of category-specific contextual details experienced at encoding (Polyn et al., 2005). This interpretation is akin to the concept of “retrieval orientation,” the adoption of goal-directed retrieval sets that influence probe processing to optimize the recovery of sought-after information (Herron and Wilding, 2006). Previous studies have shown that retrieval orientations can be initiated by instructional cues on a trial-by-trial basis, but these studies have not related such activity to retrieval success (Herron and Wilding, 2006, Johnson and Rugg, 2006). It is worth noting that the 1 study that found a relationship between preprobe retrieval activity (in the form of theta oscillations) and source retrieval used a blocked design (Addante et al., 2011), similar to the present study. It may be that preprobe activity affects source retrieval only when the same retrieval goal can be maintained across trials.

However, why would older adults engage preprobe activity to a larger degree than younger adults? It is not possible to relate the frontal ERP effect on the scalp to frontal brain regions on the basis of the current data alone given the EEG inverse problem (e.g., Grech et al., 2008). However, the frontal focus of the effect is consistent with a source in the prefrontal cortex, and considerable evidence has shown that overrecruitment of the prefrontal cortex is a feature of aging (Davis et al., 2008). A common observation in the aging literature is increased activity in especially the prefrontal cortex accompanied by poor performance. For example, increased prefrontal activation has been related to poor memory encoding (de Chastelaine et al., 2011) and poor memory retrieval (Davis et al., 2008). It has been proposed that the additional prefrontal activation is an adaptive mechanism that compensates, at least in part, for the failure of neural systems responsible for the ongoing task (de Chastelaine et al., 2011). The neural adaptation, however, may not be sufficient to elevate memory performance. This partial compensation interpretation is supported by findings that age-related brain volume reduction in the prefrontal cortex accounts for the overrecruitment of the prefrontal cortex during associative retrieval (Kalpouzos et al., 2012). These ideas fit with the present results, which showed increases in retrieval-related brain activity accompanied by decreases in overall memory performance across older participants.

Although the compensatory interpretation implies that the present effect might be age-specific, the current data do not rule out the possibility that the effect is related to poor associative memory more generally. Younger adults were more homogeneous in the brain and behavior, and their memory performance was superior compared to older adults (Fig. 3). A possible relationship between preprobe activity and memory ability is consistent with a previous study demonstrating that greater demands on preprobe processing are reflected in a sustained negative-going ERP effect over frontal scalp sites (Johnson and Rugg, 2006). It has been shown that the prefrontal cortex is selectively engaged by demands to retrieve weakly rather than strongly encoded source information (Kuo and Van Petten, 2006). Therefore, it remains possible that younger adults can engage preprobe activity to the same extent as older individuals once the task is made sufficiently difficult. Future studies are needed to examine whether the effect is an intrinsic feature of aging or related to poor performance.

## 5 Conclusion

Taken together, the present findings point to a critical role of preprobe activity in associative retrieval. In older adults, the probability that a memory probe leads to the recollection of associative information differed depending on the neural activity that leads up to the probe. This activity may be related to cognitive control, which helps to select and guide relevant mental processes during retrieval attempts. The individual differences suggest that additional neural mechanisms may be recruited by poor performers, perhaps in partial compensation for their weak memory. The data also highlight the benefit of using a technique with high temporal resolution, such as EEG, to track the time course of brain activity that relates to episodic memory and pinpoint processes that happen before and after a retrieval probe. An important question for future work is whether this effect is specific to aging or related to weak memory performance more generally. Regardless, it is clear that neural activity leading up to a retrieval probe needs to be taken into consideration when trying to understand the mechanisms of memory. The findings thus open up new avenues for understanding the mechanisms of memory and aging.

## Disclosure statement

The authors declare no conflicts of interest.
